# A Fibroblast-Derived Secretome Stimulates the Growth and Invasiveness of 3D Plexiform Neurofibroma Spheroids

**DOI:** 10.3390/cancers16142498

**Published:** 2024-07-09

**Authors:** Kyungmin Ji, George J. Schwenkel, Raymond R. Mattingly, Harini G. Sundararaghavan, Zheng Gang Zhang, Michael Chopp

**Affiliations:** 1 Department of Neurology, Henry Ford Health, Detroit, MI 48202, USA; gschwen1@hfhs.org (G.J.S.); zzhang1@hfhs.org (Z.G.Z.); mchopp1@hfhs.org (M.C.); 2 Department of Pharmacology and Toxicology, Brody Medical School at East Carolina University, Greenville, NC 27834, USA; mattinglyr21@ecu.edu; 3 Department of Biomedical Engineering, Wayne State University, Detroit, MI 48202, USA; hsundara@wayne.edu; 4 Department of Physics, Oakland University, Rochester, MI 48309, USA

**Keywords:** plexiform neurofibroma, fibroblasts, growth, invasiveness, 3D cultures

## Abstract

**Simple Summary:**

Plexiform neurofibromas in neurofibromatosis type 1 (pNF1), characterized by aggressive growth and local invasiveness, are comprised primarily of Schwann cell-derived tumor cells (*Nf1*^−/−^) and the tumor microenvironment (TME). Fibroblasts (*Nf1*^+/−^), the most abundant cell type in the TME, are known to significantly contribute to pNF1 formation, but their precise role in the subsequent tumor progression is poorly understood. Using our established three-dimensional (3D) culture models with patient-derived pNF1 cells and fibroblasts, we observed that the fibroblast-derived secretome stimulates the growth and local invasiveness of pNF1 spheroids. Depletion of small extracellular vesicles (sEVs) from the fibroblast secretome completely eliminated the paracrine effect on pNF1 spheroid growth. These results suggest that targeting paracrine interactions between pNF1 tumor cells and fibroblasts may offer a potential therapeutic approach for reducing pNF1 tumor progression.

**Abstract:**

Plexiform neurofibromas (PNs) occur in about a half of neurofibromatosis type 1 (NF1) patients and have garnered significant research attention due to their capacity for growth and potential for malignant transformation. NF1 plexiform neurofibroma (pNF1) is a complex tumor composed of Schwann cell-derived tumor cells (*Nf1*^−/−^) and the tumor microenvironment (TME). Although it has been widely demonstrated that the TME is involved in the formation of neurofibromas, little is known about the effects of the TME on the subsequent progression of human pNF1. Elucidating the molecular interactions between tumor cells and the TME may provide new therapeutic targets to reduce the progression of pNF1. In the present study, we focused on the contributions of fibroblasts, the most abundant cell types in the TME, to the growth of pNF1. To simulate the TME, we used a three-dimensional (3D) coculture model of immortalized pNF1 tumor cells (*Nf1*^−/−^) and primary fibroblasts (*Nf1^+/−^*) derived from pNF1 patients. We performed live-cell imaging of 3D/4D (3D in real-time) cultures through confocal microscopy followed by 3D quantitative analyses using advanced imaging software. The growth of pNF1 spheroids in 3D cocultures with fibroblasts was significantly greater than that of pNF1 spheroids in 3D monocultures. An increase in the growth of pNF1 spheroids also occurred when they were cultured with conditioned media (CM) from fibroblasts. Moreover, fibroblast-derived CM increased the invasive outgrowth and further local invasion of pNF1 spheroids. Interestingly, when small extracellular vesicles (sEVs) were depleted from the fibroblast-derived CM, the stimulation of the growth of pNF1 spheroids was lost. Our results suggest that fibroblast-derived sEVs are a therapeutic target for reducing the growth of pNF1.

## 1. Introduction

Neurofibromatosis type 1 (NF1) is an autosomal-dominant genetic disorder caused by loss of expression of the neurofibromin (*Nf1* gene product) tumor suppressor gene on chromosome 17 and subsequent dysregulation of RAS/mitogen-activated protein kinase (MAPK) signaling in Schwann cells (SCs) [1,2]. A clinical hallmark of NF1 is the development of a benign tumor on the dermis called a neurofibroma [1,2]. Plexiform neurofibromas often occur early in life in about half of the children with NF1 [3]. Plexiform neurofibroma with neurofibromatosis type 1 (hereafter called pNF1) can cause major morbidity including disfigurement and motor dysfunction due to its compression of nerves and organs as a result of its locally invasive nature and exceptional capacity for growth [4,5,6,7]. pNF1 in patients has about 10–15% potential to develop into malignant peripheral nerve sheath tumors (MPNSTs), which metastasize and frequently result in a fatal outcome [4,5,6,8]. The prognosis for MPNST patients is poor, with a 5-year overall survival (OS) rate of 15–66% [9,10]. Currently, the primary treatment for pNF1s is surgical removal; however, surgery can have severe consequences, such as quadriparesis. Also, there is a high rate of tumor recurrence because complete removal is often difficult due to the proximity of pNF1 to vital body structures and its high degree of infiltration [11,12,13]. Only one drug, selumetinib, has been approved by the US FDA (New Drug Application: 213756 in April 2020) [14,15] for the treatment of pediatric patients in which pNF1 cannot be removed by surgery [16]. pNF1 patients have a partial response to selumetinib, with treatment provoking a tumor volume shrinkage of ~20% in 70% of pNF1 patients [16].

Neurofibromas are complex tumors that are composed of SC-derived tumor cells (*Nf1*^−/−^) and the tumor microenvironment (TME), which include cellular components (e.g., fibroblasts, endothelial cells, and inflammatory cells) [17]. The cellular microenvironment is implicated in the development of neurofibromas through a paracrine loop between the tumor cells and the cellular microenvironment [17,18,19]. However, the contribution of the cellular microenvironment to the subsequent growth and invasiveness of existing pNF1 tumors remains unknown. Fibroblasts, the most abundant cell type in the pNF1 TME [20], produce a dense collagenous extracellular matrix (ECM), a hallmark of neurofibromas [21], and contribute to the formation of neurofibromas [22,23,24]. However, their precise roles in tumor formation and progression are still under-investigated. Therefore, delineating paracrine interactions between pNF1 cells and other cells in their microenvironment that mediate growth and invasiveness may help identify potential therapeutic targets for mono and combination therapies.

Tumor cells grown in a two-dimensional (2D) monolayer culture do not replicate *in vivo* conditions of the physiological microenvironment including the structural architecture and stroma [25,26]. This has led to the development of alternative preclinical 3D culture models in which tumor cells are grown in an ECM [25,26]. Such 3D models provide an improved translational capacity for assessing the efficacy of a wide variety of therapies [27,28,29,30] and replicating the resistance to cytotoxic therapies that occurs *in vivo* [31,32,33,34,35]. We have developed 3D/4D (3D + time) culture models of pNF1 cells for screening of therapeutic targets associated with the growth of pNF1 [36] in real-time and over a period of time (4D). Our 3D/4D pNF1 culture models can be used for live-cell imaging and molecular and biochemical analyses of interactions between tumor cells and other cell types, e.g., fibroblasts, found in the TME of pNF1. Our 3D/4D coculture models expand typical *in vitro* analyses of pathobiological pathways to include dynamic and spatiotemporal processes as live cells interact in real-time [37,38].

Here, we assessed paracrine interactions of pNF1 cells and fibroblasts in a 3D culture system. Using our 3D pNF1 culture models, the present study demonstrates that pNF1 spheroids formed by pNF1 cells in cocultures with fibroblasts exhibit a significant increase in growth when compared with monocultures. In addition, the fibroblast secretome significantly increased the growth of pNF1 spheroids. This effect was ablated when sEVs were depleted from the fibroblast secretome. Our results suggest that paracrine interactions between tumor cells and fibroblasts via fibroblast-derived sEVs may be targeted to reduce the growth of pNF1 tumors.

## 2. Materials and Methods

### 2.1. Reagents

Reduced growth factor reconstituted basement membrane (rBM; Cultrex^TM^) and Collagen I (high concentration; rat tail) were purchased from Bio-Techne (Minneapolis, MN, USA) and Corning (Corning, NY, USA), respectively. Phenol red-free Dulbecco’s modified Eagle’s medium (DMEM) and MycoZap^TM^ Plus-CL were purchased from Lonza (Basel, Switzerland). Fetal bovine serum (FBS) was sourced from Cytiva (Marlborough, MA, USA). L-glutamine, MTT (3-(4,5-dimethylthiazol-2-yl)-2,5 diplenyltetrazolium bromide) substrates, and all other chemicals, unless otherwise stated, were purchased from Sigma-Aldrich (St. Louis, MO, USA). CellTracker^TM^ Orange, CellTrace^TM^ Far red, Hoechst33342, LookOut RT-PCR kits for mycoplasma detection, and Thermo Scientific Three-Step Stain (methylene blue and eosin) were purchased from Thermo Fisher Scientific (Waltham, MA, USA). A 24-well cell culture insert (CellQART) was purchased from Sterlitech (Auburn, WA, USA). Human Inflammation Arrays were purchased from Abcam (Cambridge, MA, USA).

### 2.2. Cells and Cell Maintenance

The following immortalized human plexiform neurofibroma cell lines (hereafter called pNF1 cells) described in [36] were used: ipNF95.11b C, ipNF95.11b C-RFP, and ipNF 05.5. ipNF95.11b C gifted from Dr. Margaret Wallace (University of Florida, USA) was lentivirally transduced with red fluorescent protein (RFP) by Dr. Raymond R Mattingly’s group [36], and ipNF95.11b C and ipNF05.5 were purchased from ATCC. Primary fibroblasts (hereafter called fibroblasts) from pNF1 patients were purchased from Dr. Margaret Wallace, University of Florida (USA). All cells were maintained as monolayers at 37 °C, 5% CO_2_ in growth media (DMEM/high glucose + 10% FBS). Cells were routinely checked to ensure that they were free of mycoplasma contamination.

### 2.3. Three-Dimensional (3D) Culture

**Figure 1:**
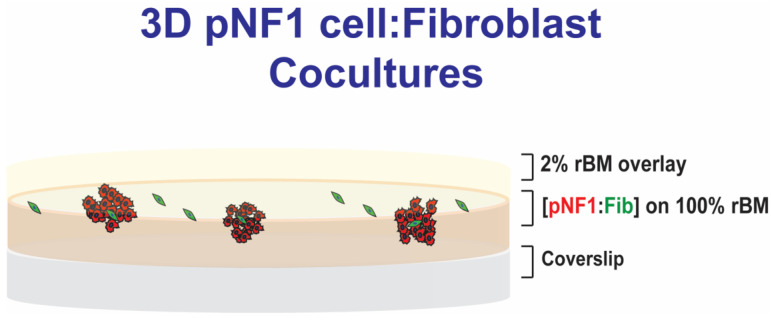
Schematic of 3D pNF1 cell:fibroblast (Fib) cocultures. pNF1 cells (8 × 10^3^ cells) and fibroblasts (2 × 10^3^ cells) at a ratio of 4:1 were plated on reconstituted basement membrane (rBM), Cultrex^TM^: Collagen I mixture, on glass-bottomed culture dishes, overlaid with DMEM with 2% FBS in 2% rBM, and grown for 6 days.

We used a 3D rBM overlay model according to previous publications for live-cell imaging by confocal microscopy [36,37]. Briefly, in 3D monocultures of pNF1 cells (8 × 10^3^ cells) or fibroblasts (2 × 10^3^ cells), cells were seeded on rBM, Cultrex^TM^: collagen I mixture, on glass-bottomed culture dishes (14 mm in diameter), overlaid with DMEM with 2% FBS in 2% rBM, and grown for 6 days, as illustrated in Figure 1. In 3D pNF1 cell: fibroblast cocultures, fibroblasts were plated first on rBM and allowed to attach before adding tumor cells. Then, pNF1 cells were placed on top of fibroblasts, followed by 2% rBM in DMEM. A seeding ratio of 4 pNF1 cells to 1 fibroblast was used for 3D cocultures. pNF1 cells were transduced with RFP or were labeled with CellTracker^TM^ orange, and fibroblasts were labeled with CellTrace^TM^ Far Red prior to seeding to distinguish each cell type in 3D cocultures. Nuclei were stained with Hoechst33342. Cell culture media were replaced with fresh 2% overlay every 4 days.

### 2.4. Image Acquisition for Quantitative Analysis in 3D

Optical sections of 9 or 16 contiguous fields, unless otherwise indicated, through the entire depth of the 3D structures were acquired using Zeiss LSM 780 (Carl Zeiss Microscopy, Jena, Germany) or Stellaris 5 (Leica Microsystems, Deerfield, IL, USA) confocal microscopes. Images were reconstructed in 3D using Volocity software 7.0.0 (PerkinElmer, Waltham, MA, USA). The number of total cells was quantified using Volocity. X (green), y (red), and z (blue) arrows in the bottom left of each image indicate the orientation of 3D images. Representative images of 3D reconstructions in each figure are shown in different angles to visualize the results more clearly. DIC images were taken using a Nikon Eclipse Ti-S microscope (Tokyo, Japan).

### 2.5. Fibroblast-Conditioned Media (CM)

Fibroblasts (1 × 10^6^ cells in a 60 mm dish) in fresh DMEM, i.e., DMEM not exposed to cells, in 2% rBM were added on top of 100% rBM and maintained for 6 days. Media were collected and centrifuged at 1800 × *g* to remove debris and floating cells, and supernatants were aliquoted to avoid damage from multiple freeze–thaw cycles. The resulting CM from fibroblasts (Fib-CM) or the cell-free condition (control media; CtrlM) was stored at −20 °C until use. For the 3D imaging experiments, pNF1 cells (8 × 10^3^ cells) were then seeded on 100% rBM, overlaid with 2% rBM, and grown in the presence of either CtrlM or Fib-CM mixed at a ratio of 1:1 with fresh DMEM; media were replaced every other day.

### 2.6. sEV Depletion from Fib-CM

Depletion of sEVs from Fib-CM was performed, as previously described [39]. Briefly, media from 6-day monocultures of fibroblasts (1 × 10^6^ cells in a 60 mm dish) in fresh DMEM were collected and centrifuged at 3000 × *g* to remove debris and floating cells, followed by 0.22 µm filtration. The Fib-CM was ultracentrifuged at 100,000 × *g* for 2 h. The supernatant was used as sEV-depleted Fib-CM. sEV depletion was determined by version 3.3 Dev Build 3.3.104 Nanoparticle Tracking Analysis software (Malvern Panalytical Ltd., Malvern, UK) installed in NanoSight NS300 (Malvern Panalytical Ltd., Malvern, UK).

### 2.7. In Vitro 3D Invasion Assay

We performed 3D invasion assays as previously described [38]. Briefly, pNF1 cells (3 × 10^3^ cells) were seeded on top of 8 μm cell culture filter inserts coated with 50 μL of 5 mg/mL rBM. The cells were allowed to form tumor spheroids for 4 days, which were then incubated for a further 24 h with either control media (CtrlM) or Fib-CM in the lower compartment. Cells on the bottom of the inserts, i.e., invaded cells, were fixed with 3.7% formaldehyde and stained with Thermo Scientific Three-Step Stain (methylene blue and eosin). After the stained filters were mounted on slides, invaded cells were visualized using a Nikon Eclipse Ti-S microscope (Tokyo, Japan) and quantified using ImageJ software (NIH, Bethesda, MD, USA). Images of seven random fields per insert were acquired, and three independent experiments were carried out.

### 2.8. 3D MTT Assay

A 3D MTT assay was used as an indicator of cell growth and viability, performed as previously described [36]. On day 0, 1.2 × 10^4^ pNF1 cells (ipNF95.11b C) were plated in 150 μL media on 100% rBM with a 2% rBM overlay (DMEM, 10% FBS) per well in triplicate or quadruplicate. Empty cells along the perimeter of the plates were filled with PBS to minimize media evaporation. Twenty-four hours after plating cells, the medium was replaced with 150 μL DMEM (5% FBS). On day 5, 20 μL of 5 mg/mL MTT solution was added to each well, and cells were incubated for 4 h until a purple-colored formazan product was formed. After incubation, the wells were carefully aspirated. The formazan product was incubated overnight at 37 °C in a 10% SDS in 0.01% HCl solution to solubilize the formazan and the rBM. The optical density (OD) was measured at a wavelength of 570 nm with the background absorbance read at 690 nm by an ELISA plate reader (SpectraMax Plus 384 microplate reader; Molecular Devices, San Jose, CA, USA).

### 2.9. Cytokine Inflammation Array

CM was obtained from 3D monocultures of pNF1 cells (ipNF95.11b C) and fibroblasts and cocultures of pNF1 cells and fibroblasts for analysis. Experiments were performed according to the manufacturer’s recommended protocols. Arbitrary values of the dot intensity representing detected cytokines were quantified by densitometry and normalized to positive controls on the same membrane using ImageJ software (v1.51w) (NIH, Bethesda, MD, USA).

### 2.10. Statistical Analysis

Data are presented in box-and-whisker plots or bar graphs as the mean ± standard deviation. In box-and-whisker plots, boxes represent interquartile ranges and whiskers the minimum and maximum values. The significance of differences was evaluated either by one-way ANOVA, followed by Tukey’s post hoc test to correct for multiple comparisons, or by Student’s *t*-test for two groups using GraphPad Prism version 8.0.1 for Windows, GraphPad Software (Boston, MA, USA). For all studies, *p* ≤ 0.05 was considered statistically significant.

### 2.11. Patents

The present study is associated with the U.S. Patent US10227556B2.

## 3. Results

### 3.1. Fibroblasts Stimulate the Growth of pNF1 Spheroids in 3D Cultures

**Figure 2:**
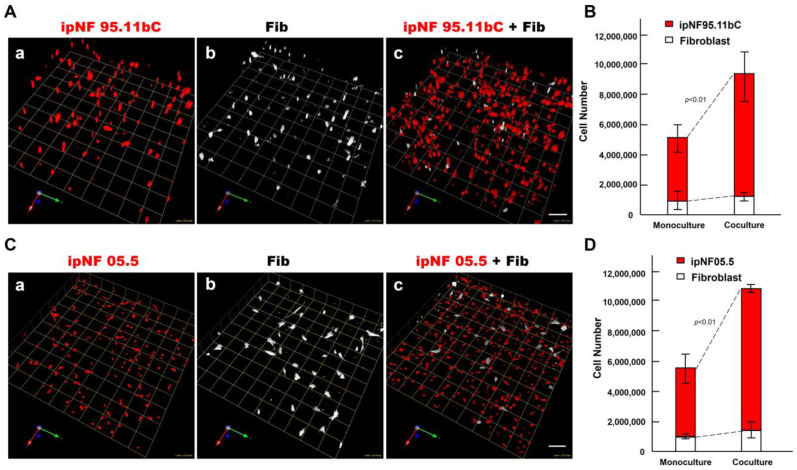
An increase in the growth of pNF1 spheroids formed by pNF1 cells was observed in the presence of fibroblasts in 6-day 3D cultures. (**A**) Representative images of 3D reconstructions of monocultures of pNF1 cells [ipNF95.11b C (**A**(**a**)), ipNF05.5 (**C**(**a**)); red] or fibroblasts (primary fibroblasts 05.2; (**A**(**b**),**C**(**b**)); pseudo-white) and pNF1 cell:fibroblast cocultures (**A**(**c**),**C**(**c**)). Images are tiled from 16 ((**A**); a grid and a scale bar, 170 μm) or 9 ((**C**); a grid and a scale bar, 128 μm) contiguous fields. (**B**,**D**) Total number of nuclei of pNF1 spheroids [ipNF95.11b C (**B**) and ipNF05.5 (**D**); red bars] and fibroblasts (white bars) when grown alone (monoculture) or in coculture were quantified in 3D with Volocity. Data are expressed as mean ± standard deviation.

**Figure 3:**
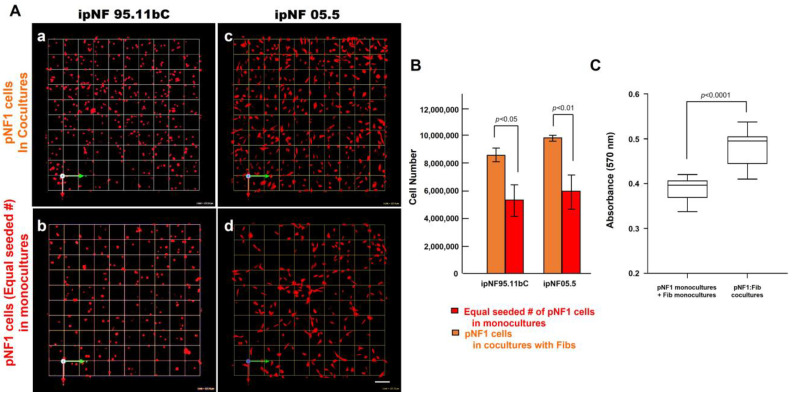
An increased growth of pNF1 spheroids resulted from coculturing them with fibroblasts over a 6-day culture period. (**A**) En face views of 3D reconstructions of pNF1 cell [ipNF95.11b C (**a**,**b**) and ipNF05.5 (**c**,**d**); red]: fibroblast cocultures (**a**,**c**) and pNF1 monocultures (**b**,**d**) at day 6. Only pNF1 spheroids are visualized in a and b. The initial cell seeding number of pNF1 cells in monocultures in b and d is the same as total number of cocultures. Images are tiled from 9 contiguous fields; each grid and a scale bar represent 128 μm. (**B**) The number of nuclei of only pNF1 spheroids was quantified in 3D with Volocity. Data are expressed as mean ± standard deviation. (**C**) Monocultures of pNF1 cells (ipNF95.11b C) or fibroblasts and cocultures of pNF1 cells and fibroblasts were incubated for 6 days. Cell growth/viability was measured using a 3D MTT assay. The OD values of cocultures were compared with ones of two monocultures, i.e., pNF1 cells alone and fibroblasts alone.

To determine the effect of fibroblasts on the growth of pNF1 tumor cells within the TME, we compared the growth of two human pNF1 cell lines, ipNF95.11b C and ipNF05.5, when cultured in the absence or presence of human fibroblasts derived from pNF1 patients. Over a 6-day culture period, pNF1 cell lines formed 3D spheroid structures in monocultures. While there was a slight increase in the number of fibroblasts in the cocultures compared to the monocultures, a significant increase in the growth of pNF1 spheroids in the cocultures was observed (Figure 2A,C). An increase in the growth of pNF1 spheroids in the cocultures was verified by 3D quantitative analysis (Figure 2B,D). This result suggests that paracrine interactions between pNF1 spheroids and fibroblasts increase the growth of pNF1 spheroids. To further validate our observations on the increased growth in pNF1 spheroids resulting from paracrine interactions with fibroblasts, we compared the growth of pNF1 spheroids between pNF1 cell: fibroblast cocultures and pNF1 monocultures, which had an equal seeding density to the cocultures. Indeed, there was a significant increase in the growth of pNF1 spheroids in the cocultures compared to the pNF1 monocultures over a 6-day culture period (Figure 3A,B). In line with our previous observations, we confirmed the increased growth of pNF1 spheroids in cocultures with fibroblasts using a 3D MTT assay. The OD value of pNF1 spheroids and fibroblasts grown in cocultures was greater than the combination of each cell type grown in monocultures (Figure 3C). Collectively, these results reveal that paracrine interactions between pNF1 spheroids and fibroblasts enhance the growth of pNF1 tumor spheroids.

### 3.2. Fibroblast-Derived Secretome Increases the Growth of pNF1 Spheroids

**Figure 4:**
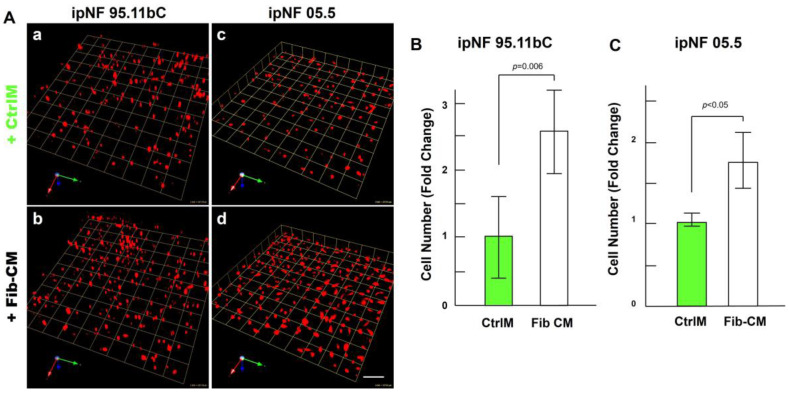
Fibroblast-derived CM increases the growth of pNF1 spheroids in 6-day 3D cultures. (**A**) Angled images of representative 3D reconstructions of monocultures of pNF1 cells (ipNF95.11b C, (**a**,**b**); ipNF05.5, (**c**,**d**); red) in CtrlM (**a**,**c**) or Fib-CM (**b**,**d**) at 6 days. Images are tiled from 9 contiguous fields; each grid and a scale bar represent 157 μm (**a**,**b**) or 128 μm (**c**,**d**). (**B**,**C**) The total number of nuclei of pNF1 spheroids with CtrlM (green bars) or Fib-CM (white bars) was quantified in 3D with Volocity. Data are expressed as mean ± standard deviation.

The observed increase in the growth of pNF1 spheroids in 3D cocultures with fibroblasts suggests that paracrine factors secreted from fibroblasts mediate tumor growth in pNF1 progression. Therefore, we investigated whether the observed increase in the growth of pNF1 spheroids with fibroblasts could be caused by paracrine secretory factors from fibroblasts. The effects of Fib-CM or control media (CtrlM) were compared in pNF1 monocultures over a 6-day culture period. The growth of pNF1 spheroids formed by ipNF95.11b C cells increased by about 2.5-fold in Fib-CM compared to those in control media (Figure 4A(a,b),B. An increase in pNF1 spheroid growth in the absence of direct contact with fibroblasts indicates that the fibroblast-derived secretome induces pNF1 cell growth via paracrine interaction. These results were consistent with ipNF05.5 cells under the same conditions. We observed about a 2-fold increase in cells in Fib-CM compared to those in control media (Figure 4A (c,d),C).

### 3.3. Fibroblast-Derived Secretome Stimulates the Growth and Invasive Outgrowth of pNF1 Spheroids

**Figure 5:**
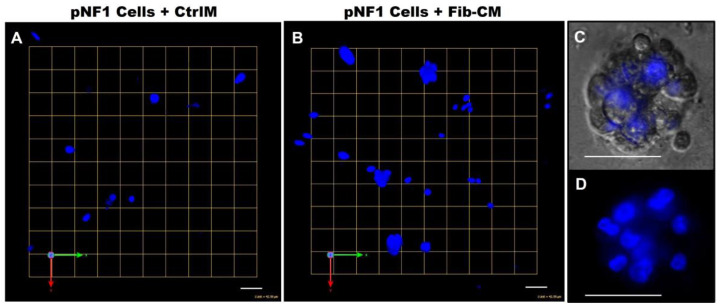
Fibroblast-derived CM increases the spheroid formation of pNF1 cells. (**A**,**B**) En face views of 3D reconstructions of Hoechst33342-stained pNF1 spheroids (blue) formed by ipNF95.11b C in CtrlM (**A**) or Fib-CM (**B**). Images are from a single field; each grid and scale bars represent 43 μm. (**C**,**D**) Enlarged DIC (**C**) and Hoechst-stained 3D images (**D**) illustrate a pNF1 spheroid in greater detail; scale bars, 43 μm.

**Figure 6:**
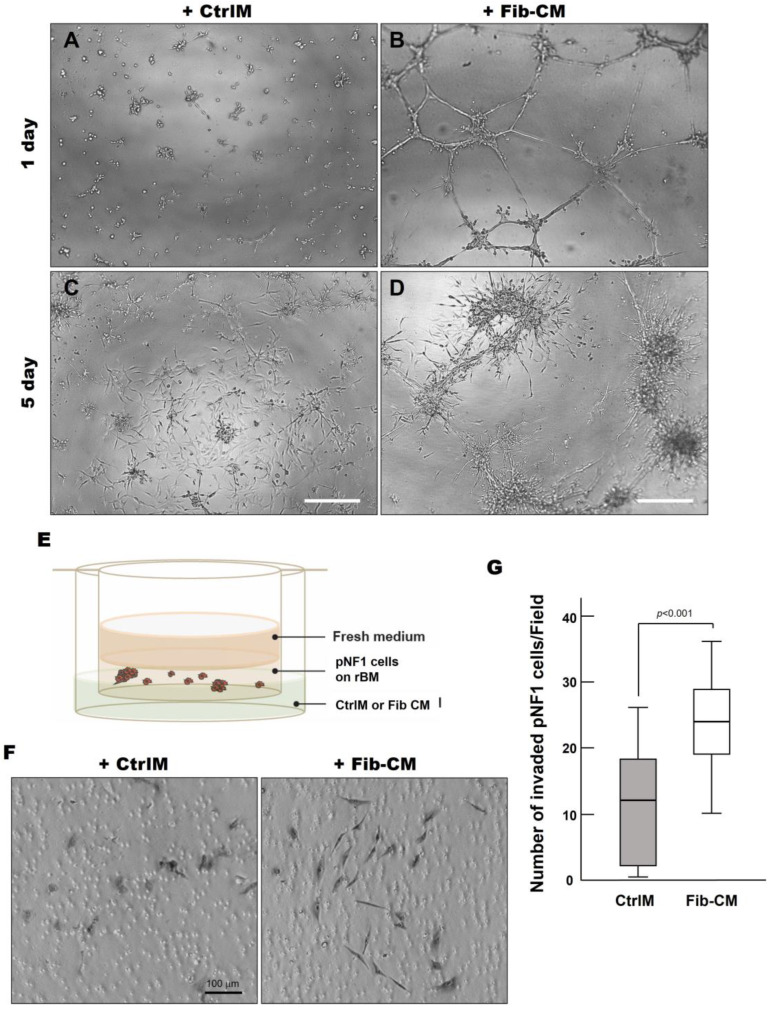
Fibroblast-derived secretome increases the invasive outgrowth and local invasion of pNF1 spheroids. (**A**–**D**) DIC images of pNF1 spheroids in CtrlM (**A**,**C**) or Fib-CM (**B**,**D**) at 1 and 5 days. mScale bars, 500 µm. (**E**) Schematic of *in vitro* 3D invasion assay: pNF1 spheroids were cultured in the presence of control media (CtrlM) or fibroblast-conditioned media (Fib-CM). (**F**) DIC images of invaded cells attached to the underside of the insert. Scale bar, 100 µm. (**G**) Invaded pNF1 spheroids incubated in the presence of CtrlM (gray bar) or Fib-CM (white bar) for 2 days were quantified with ImageJ.

To increase our understanding of the quantitative results, we sought to determine if there were differences in physical characteristics of pNF1 spheroids compared to pNF1 spheroids with Fib-CM. We performed Hoechst staining to examine the 3D structures of nuclei in pNF1 spheroids cultured in CtrlM and observed the development of large spheroid formations composed of multiple tumor cells in pNF1s cultured in Fib-CM but few in pNF1 cultured in CtrlM (Figure 5). Moreover, pNF1 spheroids cultured in Fib-CM were significantly larger than pNF1s cultured in CtrlM, and the pNF1s cultured in Fib-CM exhibited multicellular invasive outgrowths from pNF1 spheroids at day 1 (Figure 6B), whereas these outgrowths were not observed under control media conditions (Figure 6A). Images taken four days later further highlighted how the fibroblast-derived secretome promotes cell growth. pNF1 spheroids grown in Fib-CM for five days demonstrated large cell clusters connected through long cell extensions (Figure 6D). pNF1 spheroids formed in the CtrlM, however, were much smaller and lacked long invasive outgrowths (Figure 6C).

Considering the extensive outgrowths from pNF1 spheroids when incubated with Fib-CM, we performed a 3D invasion assay, as illustrated in Figure 6E, to determine the effect of the fibroblast-derived secretome on the local invasive character of pNF1 spheroids *in vivo* [13]. We observed a significant increase in the number of invasive cells exposed to Fib-CM compared to CtrlM (Figure 6F,G). In addition, the invasive pNF1 cells in the Fib-CM group were larger with longer extensions, suggesting that the morphological changes promoted by the fibroblast-derived secretome coincide with the increased invasiveness of the tumor cells (Figure 6F). These findings highlight how the fibroblast-derived secretome promotes the growth and invasiveness of pNF1 spheroids in pNF1 progression.

### 3.4. Fibroblast-Derived Small Extracellular Vesicles (sEVs), as a Paracrine Factor, Increase the Growth of pNF1 Spheroids

**Figure 7:**
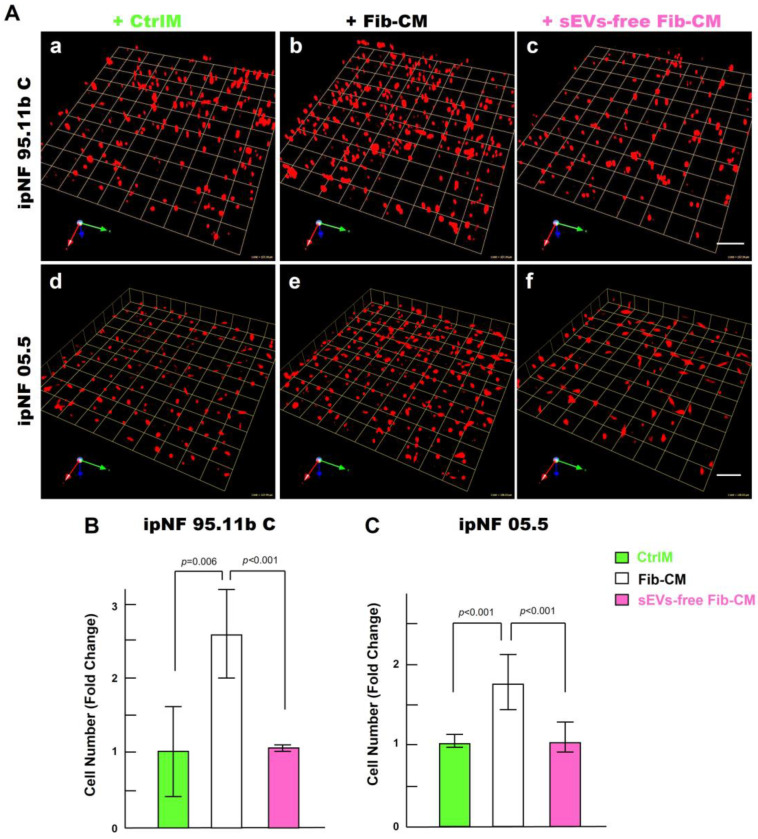
Effects of the fibroblast-derived secretome on the increase in the growth of pNF1 spheroids are abolished by sEVs depletion. (**A**) Angled views of representative 3D reconstructions of pNF1 spheroids (red) formed by ipNF95.11b C (**a**–**c**) or ipNF05.5 (**d**–**f**) in CtrlM (**a**,**d**), Fib-CM (**b**,**e**), or sEV-deprived Fib-CM (**c**,**f**) at 6 days. Images are tiled from 9 contiguous fields; each grid and scale bars represent 128 µm. (**B**,**C**) Total number of nuclei of ipNF95.11b C (**B**) or ipNF05.5 (**C**) incubated with CtrlM (green bars), Fib-CM (white), or sEVs-free Fib-CM (pink), as quantified with Volocity.

To identify paracrine factors from fibroblasts that mediate the growth of pNF1 spheroids, we first performed a cytokine immunoarray targeting known proinflammatory cytokines and growth factors. Media were collected from 3D monocultures of pNF1 cells (ipNF95.11b C), fibroblasts, and the cocultures for analysis. We observed no differences in cytokine levels in the media from all three cultures (Supplementary Figure S1). We next examined other factors that could mediate an increase in the growth of pNF1 spheroids through intercellular communication between tumor cells and fibroblasts. sEVs (30–150 nm in diameter) are released by all types of cells including tumor cells and their surrounding cells, which include fibroblasts (see a review [40]). sEVs mediate intercellular communication by transferring their cargos to recipient cells, impacting cancer cell growth and migration/invasion (see a review [40]). We initially tested the effect of depleting the Fib-CM of sEVs by ultracentrifugation, as in our published protocols (Supplementary Figure S2 and [39]). sEV-depleted Fib-CM exhibited ~ a 94% reduction in sEVs in the range of 30~150 nm. There was no growth of pNF1 spheroids when sEVs were depleted from the fibroblast-CM (Figure 7): ipNF95.11b C (Figure 7A(a–c),B) and ipNF05.5 (Figure 7A(d–f),C). These results support the premise that sEVs derived from fibroblasts act as paracrine factors to enhance the growth of pNF1 spheroids.

## 4. Discussion

Fibroblasts are the most abundant cell types in the pNF1 TME and contribute to neurofibroma formation [22,23]. A recent study by Serra’s group showed that fibroblasts enhanced the tumor formation from pNF1-induced pluripotent stem cell (iPSC)-derived SCs in a mouse model [24]. However, the underlying mechanisms that contribute to the progression of pNF1 are still poorly understood. The excessive production of collagens by fibroblasts is a hallmark of pNF1 tumors. Down-regulation of metalloproteinase (MMP) 1, the major proteolytic enzyme degrading collagen I, in fibroblasts from NF1 patients is observed [41]. The increased collagens can influence tumor growth and further progression in many solid tumors [42,43,44]; however, whether this occurs for pNF1 remains unknown. Accumulating evidence reveals that changes in fibroblast subtypes during cancer progression are closely associated with clinical outcomes [45,46,47,48]. A deeper understanding of how fibroblasts affect the progression of pNF1 is urgently needed in order to develop an effective targeted therapy for pNF1.

In this article, we have demonstrated that fibroblasts increase the growth and invasiveness of pNF1 spheroids in 3D via secretome release. The number of cells for the initial seeding density has been optimized for our 3D cultures on glass-bottomed culture dishes (14 mm in diameter) and our patented microfluidic culture devices (14 mm in diameter; Patent US10227556B2) for a short culture period of <7 days. To our knowledge, there are no reports that identify the ratios of tumor cells and fibroblasts with progression in pNF1 and for other cancers. Instead, many groups studying the roles of fibroblasts in *in vitro* cancer models employ a randomized ratio of fibroblasts and cancer cells [49,50,51]. The ratio of four tumor cells to one fibroblast in this study marks our initial approach to investigating the contributions of fibroblasts to tumor growth and invasion in our 3D pNF1 model. The ratio will likely change depending on the stage of pNF1 progression, where the biopsy is taken from, and if there is any treatment. Nonetheless, what is remarkable about our findings is how few fibroblasts are needed to affect the growth and invasiveness of pNF1 spheroids. In this study, we employed immortalized pNF1 cell lines derived from pNF1 patients since it is difficult to grow pNF1 cells directly from patients *in vitro*. However, we will use human iPSC cells [52] that are closer *to in vivo* conditions for 3D cultures in our future studies.

Small extracellular vesicles (sEVs, ~30–150 nm in size) secreted by all cell types are key mediators of intercellular communication by transferring their cell-specific cargos [e.g., proteins and microRNAs (miRNAs)] (see reviews [53,54]). sEVs have therapeutic potential to treat target cancer cells by delivering specific cargos with low immunogenicity and high stability (see reviews [53,54]). Changes in miRNAs, a class of small non-coding RNA, have been reported in pNF1 compared to dermal neurofibroma or MPNST [55,56,57,58]. Wu and Ratner’s group reported that miRNA-155 is highly expressed and mediates tumor formation and proliferation in a pNF1 mouse model [59]. To our knowledge, there are no studies investigating the effect of sEVs with their cargos on the progression of pNF1. The present study shows that sEVs may contribute to the growth of pNF1 spheroids in 3D cultures. We also found distinct expressions of miRNAs between wild-type SCs and pNF1 cells. Thus, we propose that sEVs, via their cargo transfer between tumor cells and fibroblasts, may offer a potential therapeutic target to reduce the growth and invasiveness of pNF1.

Our present study demonstrates that the fibroblast-derived secretome enhances the growth and invasiveness of pNF1 spheroids. Additionally, sEVs from the secretome may stand as a critical factor in increasing the growth of pNF1 spheroids in 3D pNF1 models. The fibroblast-derived secretome stimulates the growth of pNF1 spheroids, concomitant with an increase in the local invasion of pNF1 spheroids. Our study focused on the contributions of fibroblasts to the growth and invasiveness of pNF1s. Further studies are warranted on how other cellular constituents of the pNF1 microenvironment, such as macrophages and T cells, may contribute to the growth and invasiveness of pNF1.

## 5. Conclusions

Using our 3D plexiform neurofibroma cultures, we found that the fibroblast secretome—particularly, fibroblast-generated sEVs—plays a critical role in increasing the growth and invasive character of pNF1 spheroids. Our results provide insights on how the TME contributes to increased tumor growth in pNF1 patients. Thus, paracrine interactions between tumor cells and fibroblasts may offer a therapeutic target to reduce the growth and invasiveness of pNF1 tumors.

## Data Availability

Data are available upon request from the corresponding author.

## Supplementary Materials

The following supporting information can be downloaded at: https://www.mdpi.com/article/10.3390/cancers16142498/s1, Figure S1: Coculture of pNF1 cells and fibroblasts does not affect cytokine release; Figure S2: Depletion of sEVs in fibroblast-derived CM confirmed by Nanoparticle Tracking Analysis.
