# Supplementary material for: A Fibroblast-Derived Secretome Stimulates the Growth and Invasiveness of 3D Plexiform Neurofibroma Spheroids
Source: Cancers (Basel). 2024 Jul 9;16(14):2498. doi: 10.3390/cancers16142498 (PMC11274591; doi:10.3390/cancers16142498)
Supplement: Supplementary file 1 [file cancers-16-02498-s001.zip › cancers-3045606-supplementary.pdf]

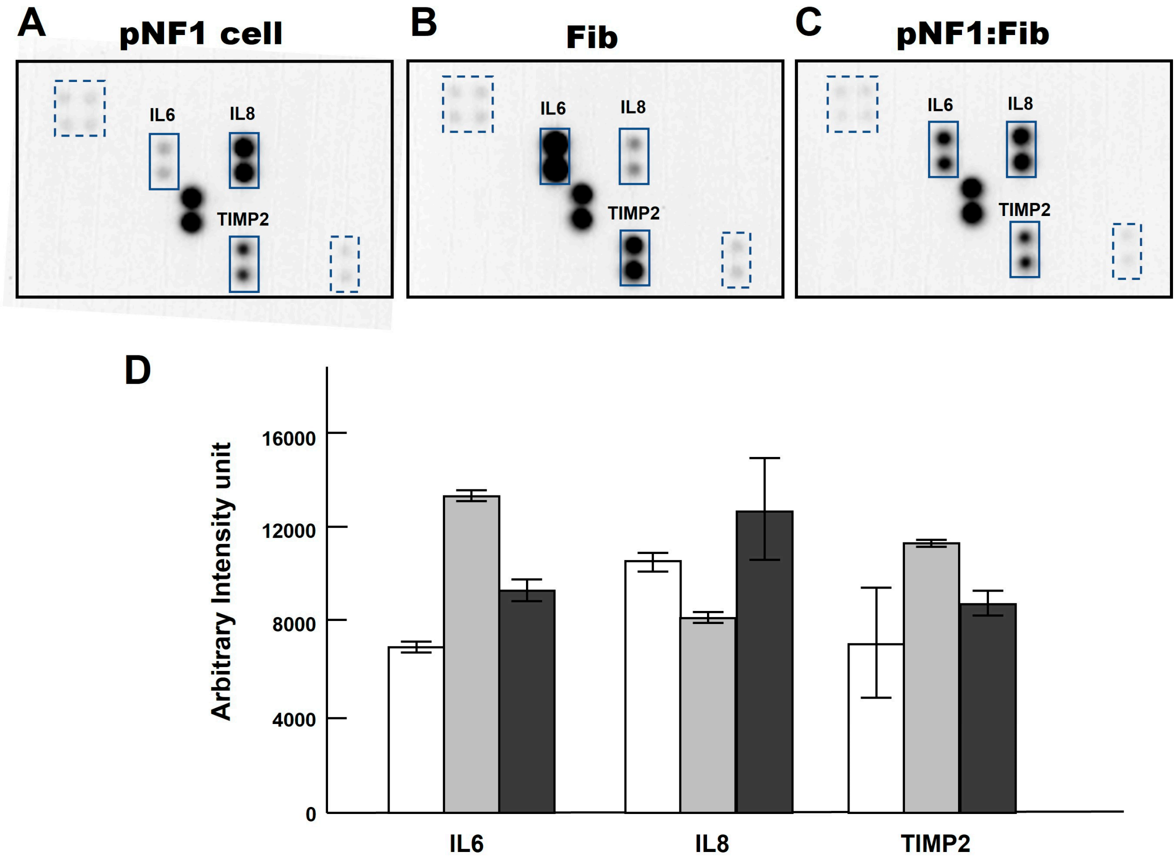

Supplementary Figure S1. Coculture of pNF1 cells and fibroblasts does not affect cytokine release. (A-C) Scan of inflammation array membrane shows spot intensities of detected cytokines and growth factors. Monocultures of pNF1 cells (ipNF95.11bC) cells (A) or fibroblasts (Fib, B) and pNF1 cell:fibroblast cocultures (C) in 3D. IL-6, IL-8, and TIMP are boxed and labeled. Positive control boxed in dashed lines next to negative controls. (D) Spot densitometry quantification of inflammation arrays for the three culture conditions: monoculture of pNF1 cell (white) and fibroblast (light gray), and pNF1 cell:fibroblast coculture (black). Results are shown as mean ± standard deviation (n=2).

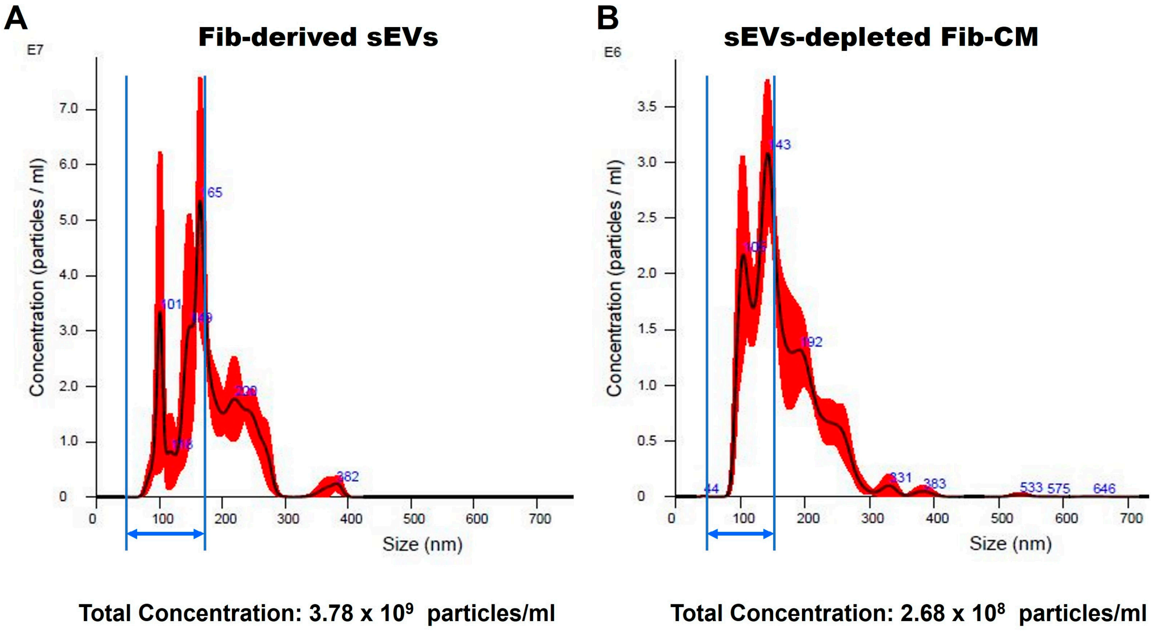

Supplementary Figure S2. Depletion of sEVs in fibroblast-derived CM confirmed by Nanoparticle Tracking Analysis. Evaluation of the size distribution (top) and the total quantity (bottom) of the sEVs from fibroblast (Fib)-derived sEVs (A) as a positive control and sEV-depleted Fib-CM (B). Note: Blue double-arrows indicate that the sizes of the sEV population range from~ 30 and ~150 nm, which is consistent with other reports (see a review, [40]).
